# Modelling the Transitioning of SARS-CoV-2 nsp3 and nsp4 Lumenal Regions towards a More Stable State on Complex Formation

**DOI:** 10.3390/ijms24010720

**Published:** 2022-12-31

**Authors:** Nele Klatte, Denis C. Shields, Clement Agoni

**Affiliations:** 1 UCD Conway Institute of Biomolecular and Biomedical Research, University College Dublin, D04 V1W8 Belfield, Ireland; 2 School of Medicine, University College Dublin, D04 V1W8 Belfield, Ireland; 3 Discipline of Pharmaceutical Sciences, School of Health Sciences, University of KwaZulu Natal, Durban 4041, South Africa

**Keywords:** SARS-CoV-2, non-structural proteins, nsp3, nsp4, transmembrane proteins, protein-protein interaction, AlphaFold2, molecular dynamics simulation

## Abstract

During coronavirus infection, three non-structural proteins, nsp3, nsp4, and nsp6, are of great importance as they induce the formation of double-membrane vesicles where the replication and transcription of viral gRNA takes place, and the interaction of nsp3 and nsp4 lumenal regions triggers membrane pairing. However, their structural states are not well-understood. We investigated the interactions between nsp3 and nsp4 by predicting the structures of their lumenal regions individually and in complex using AlphaFold2 as implemented in ColabFold. The ColabFold prediction accuracy of the nsp3–nsp4 complex was increased compared to nsp3 alone and nsp4 alone. All cysteine residues in both lumenal regions were modelled to be involved in intramolecular disulphide bonds. A linker region in the nsp4 lumenal region emerged as crucial for the interaction, transitioning to a structured state when predicted in complex. The key interactions modelled between nsp3 and nsp4 appeared stable when the transmembrane regions of nsp3 and nsp4 were added to the modelling either alone or together. While molecular dynamics simulations (MD) demonstrated that the proposed model of the nsp3 lumenal region on its own is not stable, key interactions between nsp and nsp4 in the proposed complex model appeared stable after MD. Together, these observations suggest that the interaction is robust to different modelling conditions. Understanding the functional importance of the nsp4 linker region may have implications for the targeting of double membrane vesicle formation in controlling coronavirus infection.

## 1. Introduction

The pandemic coronavirus disease 19 (COVID-19) is caused by severe acute respiratory syndrome coronavirus 2 (SARS-CoV-2) [1]. There is a pressing need to better understand the fundamentals of coronavirus biology to help tackle COVID-19 and future emerging serious coronavirus diseases, outbreaks, or pandemics.

Once a SARS-CoV-2 virion has entered a host cell, its genomic RNA (gRNA) is released into the cytosol, where it is translated by host ribosomes [2]. The gRNA comprises 14 open reading frames (ORFs), with ORF1a and ORF1b encoding 16 non-structural proteins (NSPs) that are essential for synthesising and replicating viral RNA [3]. Translating ORF1a and ORF1b produces replicase polyproteins (pp) 1a and pp1ab via a ribosomal frameshift [4,5]. nsp3 and nsp5 protease activities are responsible for cleaving pp1a and pp1ab into 16 separate NSPs [6,7]. Subsequently, nsp3, nsp4, and nsp6 assemble in the endoplasmic reticulum (ER) membrane and initiate membrane rearrangements to form double-membrane vesicles (DMVs) [8]. The NSPs then form the replication–transcription complex (RTC) to replicate and transcribe the viral gRNA, which takes place inside the DMVs [2,9]. The DMVs provide a protective microenvironment for viral RNA synthesis [2,5], while the interaction between nsp3 and nsp4 is particularly crucial for the replication of the virus [10]. 

Nsp3 is the largest of the three NSPs [11] and possesses two conserved transmembrane domains and one lumenal loop (in the ER lumen) [12,13,14]. Studies have demonstrated that nsp3 can stimulate membrane production and the expansion of existing membranes in SARS-CoV-infected cells [8]. Experiments indicate that nsp4 and nsp6 could have a regulatory effect on nsp3 [8,9]. 

**Figure 1:**
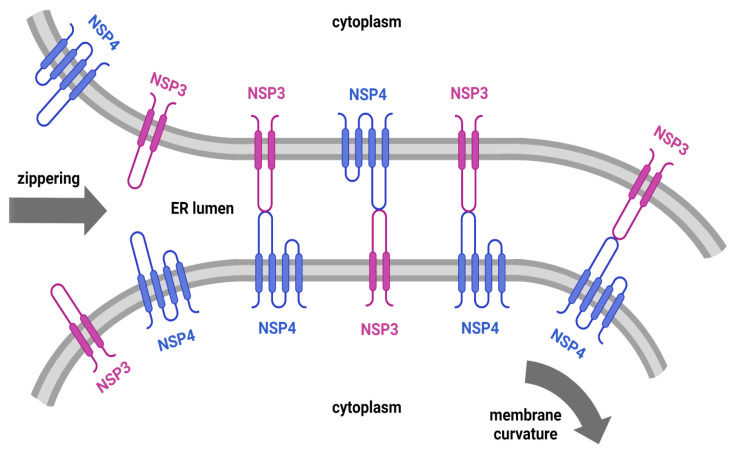
The proposed “zippering” mechanism as a model for membrane rearrangements mediated by nsp3 and nsp4. Created with BioRender.com, accessed on 30 October 2022.

Nsp4 comprises four transmembrane regions [15] and two lumenal loops, the first significantly larger than the second [13,14]. Its sequence contains an atypical glycosylation motif (NXC) [10,11]. Experimental investigations using murine coronavirus nsp4 suggest that changes in the nsp4 glycosylation site promote the formation of aberrant DMVs, indicating that nsp4 must be glycosylated to properly interact with nsp3 and induce the correct DMV formation [16]. The ER membrane extrusion needed to form DMVs is induced by the interaction between the nsp3 lumenal loop and the nsp4 large lumenal loop [17]. According to the “zippering” model, this process occurs when one NSP interacts with its counterpart on the opposite side of the ER lumen (Figure 1). This interaction acts as a zipper and induces membrane pairing and curvature, leading to DMV formation [18].

This interaction is crucial for DMV formation, where viral RNA transcription and replication occurs [17]. Hence, understanding how the nsp3 and nsp4 lumenal regions interact with each other could help develop potential drugs to disrupt their interaction and inhibit viral replication. A report by Hagemeijer et al. in 2014 demonstrated that substituting each cysteine in the large lumenal region of SARS-CoV nsp4 with serine severely affects the interaction between nsp3 and nsp4 [18]. This finding suggests that the cysteines of both lumenal regions are essential for nsp3 and nsp4 to interact by forming intra- or intermolecular disulphide bonds. High-quality protein structures can be generated using protein structure prediction software when no experimentally obtained structures are available. This study used AlphaFold2, as implemented in ColabFold [19], a promising new method for protein structure prediction that applies a deep learning technique with an AI algorithm [20]. We investigated the interaction between the nsp3 and nsp4 lumenal regions using protein structure predictions and molecular dynamics (MD) simulations. All cysteine residues in the nsp3 and nsp4 lumenal regions were modelled to be involved in intramolecular disulphide bonds, suggesting that intermolecular disulphide bonds are unlikely. We discovered a linker between two domains in the nsp4 lumenal region, which is only structured when interacting with the nsp3 lumenal region.

## 2. Results

### 2.1. Structure Prediction of the nsp3 and nsp4 Lumenal Regions

ColabFold predicted five models of the protein structure, ranked according to their predicted accuracy, and plots depicting multiple sequence alignment coverage per position, predicted aligned error (PAE), and a predicted local distance difference test (pLDDT).

The PAE is a prediction of the error in the relative positions of residues *x* and *y*. A PAE plot is a two-dimensional plot measured in Ångstrom, ranging from 0 to 30. A value of 0 indicates that the relative positions of two residues have been modelled with very high confidence [21]. Since the PAE score at position (*x*,*y*) indicates the score where *x* is the residue on which the structure is aligned, and *y* is the residue on which the error is predicted, the PAE is not necessarily symmetric.

The pLDDT was used to assess the modelled structures’ local confidence per residue and its values ranged from 0 to 100, with 100 indicating the most confidence [22].

**Figure 2:**
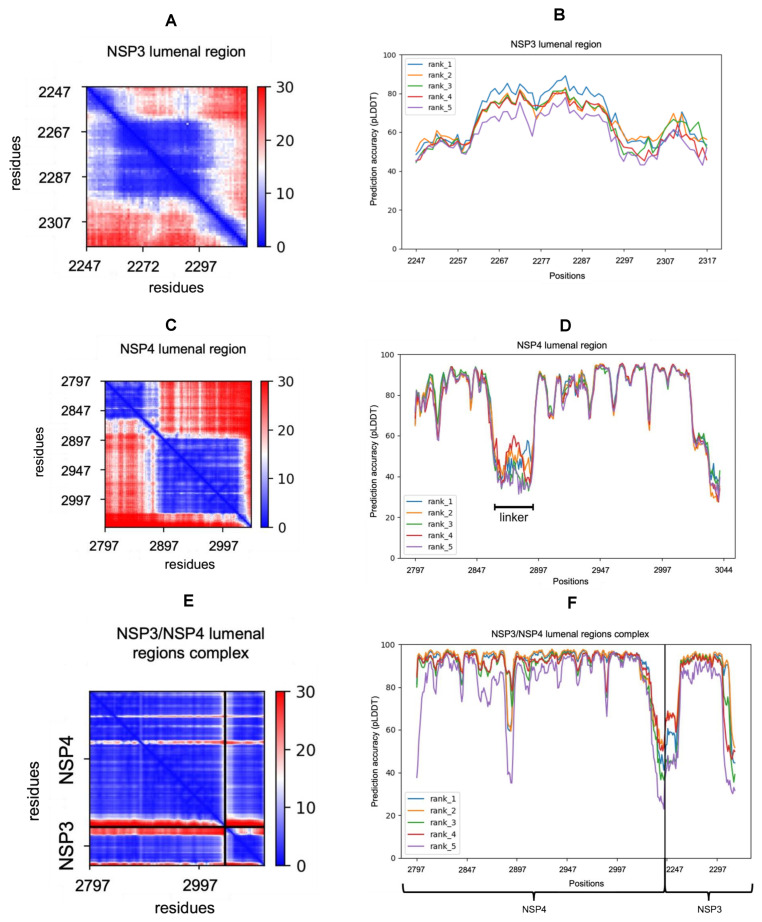
Quality assessment plots of the lumenal regions predicted alone and in complex. Blue positions in the PAE plots indicate low PAEs, red ones indicate high PAEs (measured in Angstroms). (**A**) PAE plot of the highest-ranked model of the nsp3 lumenal region. (**B**) Prediction accuracy pLDDT) plot of the five nsp3 lumenal region models (ranks 1–5). (**C**) PAE plot of the highest-ranked model of the nsp4 lumenal region. (**D**) Prediction accuracy plot of the five nsp4 lumenal region models. (**E**) PAE plot of the highest-ranked model of the nsp3 and nsp4 lumenal regions in complex. (**F**) Prediction accuracy plot of the five models of the lumenal regions of nsp3 and nsp4 in complex.

For the nsp3 larger lumenal region (residues 2260 to 2295) modelled alone (Figure 2), the N- and C-termini were modelled with less certainty (a high PAE), while the central region was modelled with more certainty (pLDDT around 80 for the highest-ranked model, dropping to 50–60 at the N- and C-termini).

For the nsp4 lumenal region, the PAE plot suggests that this falls into two “domains” (at residues 2797–2855 and 2897–3027) separated by a “linker” (2856–2986). The PAE plot suggests that error within the two domains was low, but that the relative positions of the two domains cannot be modelled accurately. These two domains were reflected in higher confidence (pled typically > 80) regions compared to the linker in nsp4 (Figure 2D). The domain pLDDT values were often lower at the positions of the cysteine residues. Conversely, the linker region had lower pLDDT values (40–50).

When the same lumenal regions of nsp3 and nsp4 were modelled together in complex, the error was greatly reduced, in particular in the linker region of nsp4 (Figure 2E). This was reflected in improved confidence in the model in the nsp4 linker region, and also in the central core of nsp3 (Figure 2F). The N- and - termini of both regions, which connect to the transmembrane helices, continued to have less certainty, indicating that their orientation in the model may well be incorrect.

**Figure 3:**
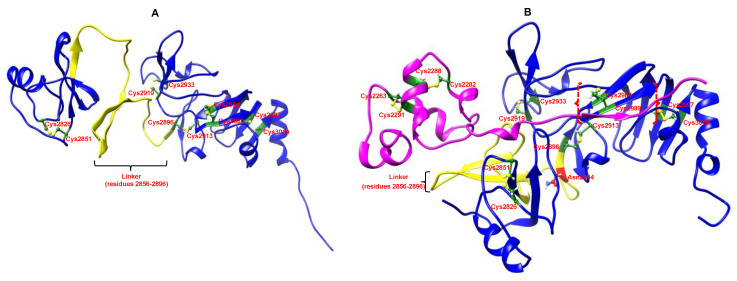
(**A**) Predicted structure of the nsp4 (blue) lumenal region (alone). Cysteine residues are highlighted in green while the linker region is coloured yellow. (**B**) nsp3 (magenta) and nsp4 (blue) lumenal regions predicted in complex with the linker region are highlighted in yellow while the cysteine residues are highlighted in green. Red curly-bracketed regions show a β-strand (residues 2309–2311) in the nsp3 lumenal region that interacts as a β-sheet with a β-strand (residues 2988–2992) in the nsp4 lumenal region. The atypical glycosylation site at Asn 2894 is coloured in red. The disulphide bonds between the cysteine residues are also highlighted in yellow.

The predicted nsp4 linker structure was characterised by β-sheets, both in the structure predicted on its own and in complex with the nsp3 lumenal region, as shown in Figure 3. Comparing the β-strand residues and their β-strand partners in both structures (Table 1 and Table 2; Figure 3), we could see that two separate beta-sheet regions (comprising two strands each) came together in the complex to form a beta structure with four strands, apparently stabilised by the nsp3 interactions. The coil region (residues 2859–2870) between the strand regions of the linker was largely exposed in the structure of nsp4 alone, but then became more buried in the complex. The two domains to either side of the linker, which were placed far apart in the nsp4 structure alone, were then brought into close contact in the complex (Figure 3).

In the modelled complex, the nsp3 lumenal region mostly contains α-helices, while the nsp4 lumenal region is characterised by β-sheet structures. The C-terminal segment of the nsp3 lumenal region primarily interacts with nsp4 (Figure 3B). Residues 2309–2311 (ITI) in nsp3 form a β-strand that interacts as a β-sheet with a β-strand in nsp4 (residues 2988–2992, CERS).

For nsp3, the same two disulphide bonds between Cys 2263 and Cys 2291 as well as Cys 2282 and Cys 2288 were observed in all of the structures. Similarly, for nsp4, the 10 nsp4 lumenal region cysteines also formed the same disulphide bonds in all of the structures (e 2826/2851, 2896/2913, 2919/2933, 2984/2989, 2997/3019). This suggests that the disulphide bonds are formed separately in the nsp3 and nsp4 lumenal regions, with no interchain disulphides (Figure 3).

Table 3 indicates the set of residue–residue interactions observed in Figure 3B between nsp3 and nsp4. Most of these represent hydrogen bonding interactions. 

### 2.2. Impact of Transmembrane Regions on Modelling

A very relevant question is whether or not the inclusion of the transmembrane regions in the model will alter the structural modelling. While AlphaFold does not consider membrane in its modelling, it is possible to include transmembrane (TM) regions in the models. Under the current zipper model of nsp3–nsp4 interaction from opposing membranes, the ideal structural complex model would project the two nsp3 TM helices into a horizontal plane opposite the plane in which the nsp4 TM helices are projected. However, since hydrophobic TM helices may tend to naturally associate in a model, we had to consider the possibility that some models of TM helices might represent artefacts arising from this tendency (e.g., with nsp3 helices complexing with nsp4 helices, contrary to the two membrane model of interaction). Accordingly, we inspected a number of different models, namely, the nsp3 lumenal region with both flanking TMs, the nsp4 lumenal region with both flanking TMs (Figures S1 and S2), and nsp3/nsp4 complexes with either the nsp3, nsp4, or both nsp3 and nsp4 TM regions (Figures S3–S5).

For nsp4 alone with TMs added in, there was some improvement in modelling confidence and a reduction in error for the linker region, but not in all models shown (Figure S1A,B). For nsp3 alone with TMs, there was increased confidence in the N terminal lumenal region (Figure S2A,B). When the nsp3 and nsp4 proteins were modelled in complex, different effects were observed from adding TMs. Adding only nsp3 TMs appeared to slightly disimprove the complex confidence, while adding the TMs of nsp4, or of both nsp3 and nsp4, gave models of somewhat improved or equivalent confidence. Some of these models are unlikely to be realistic in the exact orientation of the TM regions (Figure S5A shows a TM region interaction with the nsp3 non-TM alpha helix, which seems unlikely; S3A suggests interactions between the nsp3 and nsp4 TM regions), while others provide a better suggestion of likely orientation (Figure S4A with the helices of nsp3 projecting into a common plane, while the nsp4 termini could be reoriented in their high error termini to adopt an orientation into an opposing membrane). However, most interestingly, the core nsp3–nsp4 interactions observed in the lumenal complex model without TM regions were largely conserved in the various models including TMs (Table 3). This indicates that the modelling of the lumenal complex interactions is relatively robust to various conformational alternatives for the TMs and appear consistent with the current model of nsp3 and nsp4 interacting from opposing membranes.

Finally, extending the protein complex to include nsp6 did not markedly improve the structural conformation of nsp3 and nsp4 (Figure S2).

### 2.3. MD Simulations

Our goal in MD simulation was not to evolve the structure towards a more realistic true structure, but more simply to determine whether the core elements of the AlphaFold predicted complex appeared relatively stable in the face of MD minimisation. The starting poses for MD were taken from the AlphaFold predictions. We selected the lowest potential energy structures from across the MD simulation for further analysis and compared them with the respective pre-MD structures of the predicted structures either in complex or alone.

For the nsp3 lumenal region, the structure with the lowest potential energy was found after a simulation time of 77 ns (Table S1), for the nsp4 lumenal region after 150 ns (Table S2), and for the complex of both lumenal regions after 85 ns (Table S3).

**Figure 4:**
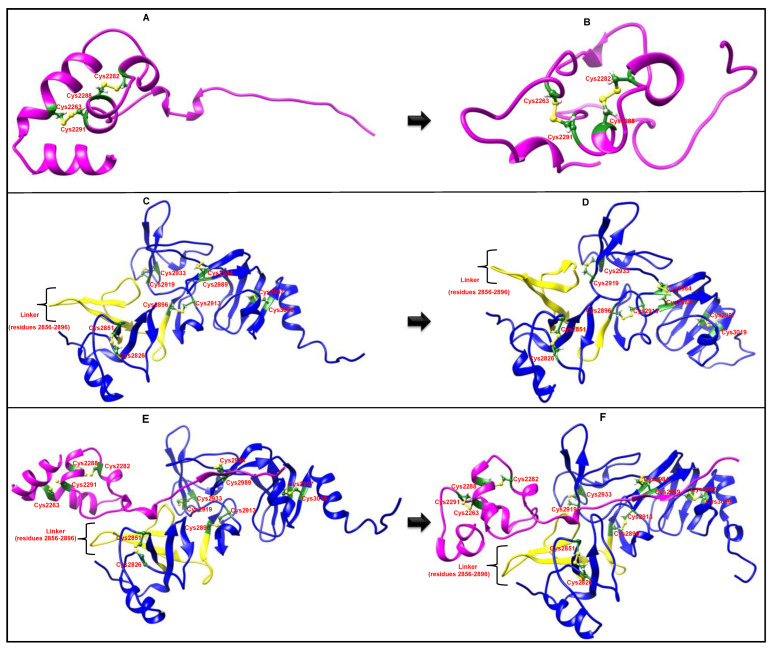
Comparison of the nsp3 and nsp4 lumenal regions either in complex or alone and their energetically most favourable structures after MD simulation. (**A**) Initial structure of nsp3 predicted alone with ColabFold prior to MD simulation. (**B**) The 77 ns energetically most favourable structure of the nsp3 lumenal region predicted with ColabFold. (**C**) Structure of the nsp4 lumenal region predicted alone. (**D**) The 150 ns MD simulation structure of the nsp4 lumenal region predicted alone. (**E**) Pre-MD structure of the nsp3/nsp4 lumenal regions predicted with ColabFold in complex. (**F**) The 85 ns MD simulation structure of the nsp3/nsp4 lumenal regions predicted with ColabFold in complex. nsp3 is shown in magenta and nsp4 is shown in blue. The linker region in the nsp4 lumenal region is coloured in yellow. Cysteine residues are highlighted in green.

Figure 4 depicts the difference between the initial structures of the nsp3 and nsp4 lumenal regions when predicted alone or in complex and their corresponding lowest potential energy structures during the MD simulations. In the case of the nsp3 lumenal region when predicted alone, the C-terminal end was stretched out initially and away from the centre of the protein (Figure 4A). However, following 77 ns of simulation, it folded close to the remaining residues, which resulted in a more compact structure (Figure 4B). In addition, it lost all of its secondary structural elements during MD simulation, indicating that the ColabFold model of nsp3 alone is not inherently stable. In contrast, it appeared more stable when modelled in complex with nsp4. The RMSD and radius of the gyration plots (Figures S6 and S8) corroborate these observations, with both showing strong fluctuations during the first 55 ns until a stable conformation was reached during the last 45 ns. Overall, the radius of gyration decreases during the simulation. The RMSF plot per residue also revealed high flexibility in the C-terminal region residues.

The nsp4 lumenal region was much more stable: before and after 150 ns of MD simulation (Figure 4C,D), the structure only underwent minor changes, and most of its secondary structure was preserved. The RMSD plot indicates that after about 110 ns, the structure was stably folded, while the radius of gyration barely changed throughout the entire simulation (Figure S7). The residues of the nsp4 lumenal region generally exhibited little flexibility during the MD simulation, with only the last 12 C-terminal residues displaying high RMSFs (Figure S7).

Figure 4E presents the structure of the predicted complex of the nsp3 and nsp4 lumenal regions prior to MD simulation whereas Figure 4F shows the lowest potential energy snapshot of the predicted complex after 85 ns MD simulation. As shown in Figure 4E,F, there were few structural changes observed in the nsp4 lumenal region before (Figure 4E) and after (Figure 4F) the MD simulation. However, a clearer difference was visible between the pre-MD (Figure 4E) and post-MD (Figure 4F) structures of the nsp3 lumenal region, as most of its α-helices were lost during the MD simulation. Its C-terminus continues to interact with the nsp4 lumenal region throughout the simulation and seems to be unaffected by the changes. Both the RMSD and the radius of the gyration plot for the nsp3 and nsp4 lumenal regions predicted in complex (Figure S8) showed that the structure was in a stable conformation between 25 ns and 85 ns of the MD simulation. The changes across the two regions alone and in complex during MD are summarised in Table 4. Overall, complex formation supports a more stable structure for both nsp3 and nsp4. A limitation of these models is that they are performed without the transmembrane anchoring. Thus, it may be most accurately summarised that the MD simulation revealed that the nsp3 lumenal region modelled alone does not appear to be an inherently stable structure in the absence of other stabilising components (nsp4 interaction or possibly transmembrane anchoring).

## 3. Discussion

We presented a structural model for the interaction of SARS-CoV-2 lumenal regions of the non-structural proteins nsp3 and nsp4, which play a key role in double membrane vesicle formation. The model suggests that the nsp4 lumenal region falls into two structural domains, separated by a flexible linker. These domains may be of uncertain relative orientation prior to nsp3 binding, but on complex formation, the linker region adopts a more stable structure that is predicted with greater confidence. The robustness of this model was suggested both from MD minimisation and from investigations of the sensitivity of the core nsp3–nsp4 interactions to the inclusion/exclusion of various TM helices.

This could be consistent with the linker being disordered in the absence of nsp3, since some disordered regions in proteins are only structured in the presence of their interaction partner [23]. However, the linker is not predicted to be highly disordered [24,25], likely because it is relatively hydrophobic. Neither is the nsp3 lumenal region predicted to be disordered. It is possible that the structural state of nsp4 in the absence of nsp3 is stabilised in vivo by other interaction partners.

Our complex model indicates that the linker in the nsp4 lumenal region primarily interacts with the extended C-terminus of the nsp3 lumenal region, but that the extended C-terminus also forms interactions with other parts of nsp4. This interaction appeared stable following MD simulation. Each lumenal region structure modelled under different conditions featured the same disulphide bonds, all of which were intramolecular. Thus, it seems unlikely that intermolecular disulphide bonds are part of the interaction between nsp3 and nsp4. The importance of the cysteines for nsp3–nsp4 interaction [18] may therefore reflect their role in stabilising the tertiary structure of each protein, allowing them to form appropriate interactions.

There are some limitations to the modelling presented. Ideally, full MD simulations with two lipid bilayer membranes would need to be performed, which additionally allowed for potential changes in membrane curvature during modelling. This would be extremely challenging to model in depth. Our model provides a preliminary indication of the likely interaction between the nsp3 and nsp4 lumenal regions. We noted that the core interactions in the model were robust to various modelling alternatives. In particular, when including or excluding different TM components, almost all sidechain interactions from the complex without transmembrane regions were also present in the complexes with transmembrane regions. This suggests that the model is relatively reliable, and not overly sensitive to particular details of the modelling environment. The model failed to predict with any clarity the precise structure in the immediately membrane proximal regions, but given that uncertainty, it was not inconsistent with a conformation in which the two proteins are embedded in opposing membranes.

A visual comparison of the nsp3 lumenal region before and after MD simulation revealed that the structure underwent significant changes during 100 ns of MD simulation. It became more compact and lost several of its secondary structure elements. Once a stable conformation was reached, the RMSD fluctuations significantly decreased, as did fluctuations in the radius of gyration. In particular, the C-terminal residues of the nsp3 lumenal region featured high RMSFs, which is consistent with the visual observation that the extended C-terminus approached the main domain during MD simulation, resulting in an overall lower radius of gyration. The significantly lower RMSD and RMSF of the nsp4 lumenal region may be due to conformational stabilisation by its five disulphide bonds. Most of the nsp4 lumenal region’s secondary structure elements were preserved during MD simulation. Only 12 C-terminal residues featured noticeably high flexibility, while the linker region of the complex appeared relatively stable during MD. 

On complex formation, the average RMSF of the nsp3 lumenal region decreased from 5.23 Å to 2.4 Å, while that of the nsp4 lumenal region was reduced from 2.19 Å to 1.56 Å. Although the nsp3 lumenal region lost most of its α-helix structures, the extended C-terminal region did not become closer to the main domain during simulation. Rather, it remained stretched because it was largely involved in the interaction.

Modelling and simulating the nsp3 and nsp4 lumenal regions in complex provided interesting insights for potential drug design to disrupt nsp3–nsp4 interaction. However, it should be noted that it was not possible to model the influence of nsp4 glycosylation at Asn 2894 [10,11], on the interaction with nsp3. The glycosylation site is located in the linker region of the nsp4 lumenal region. Therefore, the glycans attached to Asn 2894 are likely involved in the interaction between the lumenal regions. 

A recent study interpreted, from the modelling results, that nsp3 and nsp4 lumenal interaction occurred side by side in the membrane rather than on opposing membranes [26]. However, their models included transmembrane regions, which may be prone to artefactual co-association in AlphaFold prediction. Their model [26] did not identify the structural stabilisation of the linker region identified in our study and did not highlight the intramolecular disulphide bonding pattern. Our study is consistent with the zippering mechanism proposed by Hagemeijer et al. [18], which assumes that nsp3 and nsp4 are located in different ER membranes or far apart in the same membrane. Hence, in this model (Figure 1), the lumenal regions face each other in the lumen, and their interaction causes membrane pairing.

In conclusion, our study identified a linker region in nsp4 likely to play a key role in nsp3–nsp4 complex formation. It supports a model in which the cysteine residues (which are critical for complex formation [18]) play a role in determining the structural topology of the individual proteins via disulphide bonds, but that the disulphide bonds are not altered on complex formation. 

## 4. Materials and Methods

### 4.1. Structure Prediction of the nsp3 and nsp4 Lumenal Regions

Since AlphaFold2 (DeepMind, London, UK) requires a lot of storage space, this study used ColabFold [19] for protein structure prediction because ColabFold does not need to be run locally. The use of ColabFold in this study also stems from ColabFold’s ability to produce predictions that match AlphaFold2 on CASP14 targets and matches AlphaFold-multimer on the ClusPro4 dataset in prediction quality [19].

The default settings were used for predicting the nsp3 and nsp4 lumenal region structure with ColabFold. Initially, only the sequences of the nsp3 (71 residues) and nsp4 (248 residues) lumenal regions were entered as input sequences into ColabFold. Additional input options were applied to improve the prediction quality and confidence. These additional inputs included adding the adjoining transmembrane regions to the input sequences, which resulted in inputs of 113 residues for nsp3 and 290 for nsp4.

Furthermore, ColabFold’s ability to predict protein complexes was utilised by entering different combinations of protein sequences as input. First, the nsp3 and nsp4 lumenal regions were predicted in complex by separating their sequences with a colon. Next, the nsp3, nsp4, and nsp6 lumenal regions were predicted in complex because it is known that all three proteins interact to initiate DMV formation. For nsp6, the second and largest lumenal region was used for the complex prediction.

To investigate the influence of the adjoining transmembrane regions on the interaction between the nsp3 and nsp4 lumenal regions, three other variants of the nsp3 and nsp4 lumenal regions complex were modelled. The first input included the adjoining transmembrane regions of both lumenal regions (in addition to the lumenal regions); the second input only additionally included the adjoining transmembrane regions of the nsp3 lumenal region; and the third input only the ones of the nsp4 lumenal region.

Table 5 contains an overview of the Uniprot identifier and sequence range of the input sequences used for protein structure prediction with ColabFold. The complete input sequences of the individual protein fragments are presented in Table S4.

Based on the pLDDT and PAE plots generated by ColabFold, the obtained structures were assessed for quality to select the structures suitable for use in subsequent MD simulations. The residue sidechain interactions between the nsp3 and nsp4 lumenal regions were analysed with the WHAT IF Web Interface [27] and YASARA 20.10.4 [28].

Before running the MD simulations, the quality of the selected structures was improved by conducting three energy minimisations on each. The energy minimisations were carried out using YASARA 20.10.4 [28] to remove bumps and correct the covalent geometry. First, a simulation box was generated. The NOVA force field [29] was applied using a 10.5 Å force cut-off. A short steepest-descent minimisation was used to reduce conformational stress, followed by simulated annealing using a time step of 2 fs and atom velocities scaled down by 0.9 every 10th step. The simulated annealing continued until the energy improved by less than 0.05 kJ/mol per atom during 200 steps, indicating convergence.

### 4.2. MD Simulations

MD simulations were performed on three separate systems using GROMACS 2019.3 [30,31,32] on the ICHEC (Irish Centre for High-End Computing) and Sonic HPC (High Performance Computing) computer clusters supported by graphics processing unit accelerators.

The first simulated system included the nsp3 lumenal region alone, taken from the predicted complex of the nsp3 and nsp4 lumenal regions. Next, the nsp4 lumenal region on its own, taken from the same complex structure, was simulated. Finally, MD simulation was conducted for the predicted nsp3 and nsp4 lumenal regions complex. In all three systems, the TM regions were not included.

The MD simulations were conducted using the all-atom OPLS (Optimised Potentials for Liquid Simulations) force field [33,34] for 100 ns in the case of the nsp3 lumenal region and the complex and 150 ns for the nsp4 lumenal region because it required more time to converge. The files provided by Justin A. Lemkul in his GROMACS tutorial “Lysozyme in Water” [35,36] were used as input Molecular Dynamics Parameters (MDP) files for the preparation steps with minor changes to the temperature and the simulation time. 

A cubic shaped simulation box filled with water molecules as a solvent was generated to prepare the structures for MD simulation. Na^+^ and Cl^-^ ions were added to neutralise charged residue side chains. Next, a steepest-descent energy minimisation was conducted until a maximum force of 1000 kJ/mol/nm was reached. Periodic boundary conditions in all three dimensions and the Verlet algorithm [37] as a cut-off scheme were applied. The particle mesh Ewald method [38] was applied for long-range electrostatic interactions. A 1.0 nm cut-off was used for short-range van der Waals and electrostatic interactions. Potential energy energies of the structures during the energy minimisations were plotted to ascertain that the structures generated were energetically stable. The plotted potential energies of the structures during the energy minimisations are presented in Figure S9.

The Verlet algorithm [37], periodic boundary conditions, and cut-off settings were also applied in the subsequent steps. Next, equilibration was performed under the NVT ensemble for 100 ps with 2 fs time steps to stabilise the temperature, set to 310 K (human body temperature). For the NVT equilibration and the subsequent steps, the constraint algorithm LINCS [39,40] was used to reset bonds to their correct lengths. A modified Berendsen thermostat [41] was utilised for temperature coupling while the pressure coupling was off. The second equilibration step was carried out under the NPT ensemble for 100 ps with 2 fs time steps as a continuation of the previous NVT simulation to stabilise the pressure at one bar. Temperature coupling and pressure coupling were applied using the Parrinello–Rahman barostat [42]. 

Once these steps had been completed, the MD simulation was run for 100 ns or 150 ns, with 2 fs time steps, as a continuation of the NPT simulation. 

The built-in commands “rmsd”, “rmsf”, and “gyrate” were used to generate RMSD, RMSF, and the radius of gyration plots for MD simulation analysis. These known descriptors of structural stability were calculated to provide preliminary insights into the conformational stability of the predicted models over the simulation period.

Furthermore, the MD simulation trajectories were analysed to identify the most energetically favourable structures in the nsp3 and nsp4 lumenal regions for comparison with the starting structures. A systematic analysis of the trajectories was conducted for the individually simulated lumenal regions as well as the lumenal regions complex.

Multiple energy minimisations were carried out for the MD simulation structures in intervals of 10 ns using YASARA 20.10.4 [28], as described in Section 4.1. Each structure underwent energy minimisation until an all-atom minimisation RMSD below 0.05 Å was reached. The same method was applied at intervals of 2 ns and 5 ns to detect a structure with lower potential energy, starting from the structure with the lowest potential energy. This approach does not lead to absolute values for potential energies, but comes very close to the actual values.

## Figures and Tables

**Table 1:** The β-strands and their partners in the linker (residues 2856–2896) of the nsp4 lumenal region predicted alone using ColabFold.

β-Strand in the Linker	β-Strand Partner
Leu 2853–Ile 2858: LIAAVI	Ile 2806–Asp 2813: IIGYKAID
Gly 2871–Thr 2876: GTILRT	Gly 2879–Phe 2884: GDFLHF
Gly 2879–Phe 2884: GDFLHF	Gly 2871–Thr 2876: GTILRT
Ile 2895–Tyr 2897: ICY	Leu 2902–Glu 2904: LIE

**Table 2:** The β-strands and their partners in the linker (residues 2856–2896) of the nsp4 lumenal region predicted in complex with the nsp3 lumenal region using ColabFold.

β-Strand in the Linker	β-Strand Partner
Leu 2853–Ile 2858: LIAAVI	Ile 2806–Asp 2813: IIGYKAID Phe 2881–Pro 2886: FLHFLP
Thr 2872–Arg 2875: TILR	Phe 2881–Pro 2886: FLHFLP
Phe 2881–Pro 2886: FLHFLP	Thr 2872–Arg 2875: TILR Leu 2853–Ile 2858: LIAAVI
Asn 2894–Tyr 2897: NICY	Leu 2902–Tyr 2905: LIEY

**Table 3:** Residue sidechain interactions between the nsp3 lumenal region and the nsp4 lumenal region (both without transmembrane regions). For each sidechain interaction, a tick (✓) in one of the three columns on the right indicates if it is also present in one of the models of the nsp3 and nsp4 lumenal regions complexed with and without transmembrane regions.

Interaction Type	nsp3 Lumenal Region Residue	nsp4 Lumenal Region Residue	nsp3 with TMs + nsp4 with TMs	nsp3 with TMs + nsp4 without TMs	nsp3 without TMs + nsp4 with TMs
Hydrogen bond	Asn 2275	Arg 2875	✓	✓	✓
	Asp 2296	Lys 2830	✓		✓
	Asp 2296	Phe 2881	✓	✓	✓
	Ser 2303	Cys 2851		✓	✓
	Ser 2303	Gly 2814	✓	✓	✓
	Ser 2303	Gly 2815	✓	✓	✓
	Gln 2308	Ile 2961	✓	✓	✓
	Gln 2308	Arg 2985	✓	✓	
	Ile 2309	Cys 2989	✓		✓
	Ile 2309	Gly 2987	✓	✓	✓
Ionic interaction	Asp 2296	Lys 2830	✓	✓	✓
Pi–pi interaction	Tyr 2270	Phe 2881		✓	
Cation-pi interaction	Tyr 2270	Arg 2875	✓	✓	✓
Hydrophobic interaction	Leu 2298	Ile 2873	✓	✓	✓

**Table 4:** Comparison of the average RMSD of the protein backbone, the average radius of gyration, and the average RMSF per residue for each MD simulation.

Structure	Average RMSD	Average Radius of Gyration	Average RMSF
nsp3 lumenal region	9.98 Å	13.9 Å	5.23 Å
nsp4 lumenal region	4.71 Å	21.8 Å	2.19 Å
nsp3 and nsp4 lumenal regions complex	5.11 Å	22.93 Å	nsp3 lumenal region: 2.4 Å
			nsp4 lumenal region: 1.56 Å

**Table 5:** Overview of the input sequences used for protein structure prediction with ColabFold.

Description	Uniprot Identifier	Sequence Range
nsp3 lumenal region	P0DTD1	2247–2317
nsp4 lumenal region	P0DTD1	2797–3044
nsp3 and nsp4 lumenal regions	P0DTD1	2247–2317; 2797–3044
nsp3 lumenal region with adjoining transmembrane regions	P0DTD1	2226–2338
nsp4 lumenal region with adjoining transmembrane regions	P0DTD1	2776–3065
nsp3, nsp4 and nsp6 lumenal regions	P0DTD1	2247–2317; 2797–3044; 3656–3673
nsp3 lumenal region with adjoining transmembrane regions and nsp4 lumenal region with adjoining transmembrane regions	P0DTD1	2226–2338; 2776–3065
nsp3 lumenal region with adjoining transmembrane regions and nsp4 lumenal region	P0DTD1	2226–2338; 2797–3044
nsp4 lumenal region with adjoining transmembrane regions and nsp3 lumenal region	P0DTD1	2776–3065; 2247–2317

## Data Availability

Not applicable.

## Supplementary Materials

The supporting information can be downloaded at: https://www.mdpi.com/article/10.3390/ijms24010720/s1.

## References

[1] (2020). WHO Director-General’s Opening Remarks at the Media Briefing on COVID-19. https://www.who.int/director-general/speeches/detail/who-director-general-s-opening-remarks-at-the-media-briefing-on-covid-19---11-march-2020.

[2] Malone B., Urakova N., Snijder E.J., Campbell E.A. (2022). Structures and functions of coronavirus replication–transcription complexes and their relevance for SARS-CoV-2 drug design. Nat. Rev. Mol. Cell Biol..

[3] Lu R., Zhao X., Li J., Niu P., Yang B., Wu H., Wang W., Song H., Huang B., Zhu N. (2020). Genomic characterisation and epidemiology of 2019 novel coronavirus: Implications for virus origins and receptor binding. Lancet Lond. Engl..

[4] Mousavizadeh L., Ghasemi S. (2021). Genotype and phenotype of COVID-19: Their roles in pathogenesis. J. Microbiol. Immunol. Infect..

[5] V’kovski P., Kratzel A., Steiner S., Stalder H., Thiel V. (2021). Coronavirus biology and replication: Implications for SARS-CoV-2. Nat. Rev. Microbiol..

[6] Serrano P., Johnson M.A., Chatterjee A., Neuman B.W., Joseph J.S., Buchmeier M.J., Kuhn P., Wüthrich K. (2009). Nuclear Magnetic Resonance Structure of the Nucleic Acid-Binding Domain of Severe Acute Respiratory Syndrome Coronavirus Nonstructural Protein 3. J. Virol..

[7] Stobart C.C., Sexton N.R., Munjal H., Lu X., Molland K.L., Tomar S., Mesecar A.D., Denison M.R. (2013). Chimeric exchange of coronavirus nsp5 proteases (3CLpro) identifies common and divergent regulatory determinants of protease activity. J. Virol..

[8] Angelini M.M., Akhlaghpour M., Neuman B.W., Buchmeier M.J. (2013). Severe acute respiratory syndrome coronavirus nonstructural proteins 3, 4, and 6 induce double-membrane vesicles. mBio.

[9] Santerre M., Arjona S.P., Allen C.N., Shcherbik N., Sawaya B.E. (2021). Why do SARS-CoV-2 NSPs rush to the ER?. J. Neurol..

[10] Sakai Y., Kawachi K., Terada Y., Omori H., Matsuura Y., Kamitani W. (2017). Two-amino acids change in the nsp4 of SARS coronavirus abolishes viral replication. Virology.

[11] Gorkhali R., Koirala P., Rijal S., Mainali A., Baral A., Bhattarai H.K. (2021). Structure and Function of Major SARS-CoV-2 and SARS-CoV Proteins. Bioinforma. Biol. Insights.

[12] Neuman B.W. (2016). Bioinformatics and functional analyses of coronavirus nonstructural proteins involved in the formation of replicative organelles. Antiviral Res..

[13] Rep-Replicase Polyprotein 1ab-Severe Acute Respiratory Syndrome Coronavirus 2 (2019-nCoV)|UniProtKB|UniProt. https://www.uniprot.org/uniprotkb/P0DTD1/entry.

[14] (2021). UniProt Consortium UniProt: The universal protein knowledgebase in 2021. Nucleic Acids Res..

[15] Oostra M., te Lintelo E.G., Deijs M., Verheije M.H., Rottier P.J.M., de Haan C.A.M. (2007). Localization and Membrane Topology of Coronavirus Nonstructural Protein 4: Involvement of the Early Secretory Pathway in Replication. J. Virol..

[16] Gadlage M.J., Sparks J.S., Beachboard D.C., Cox R.G., Doyle J.D., Stobart C.C., Denison M.R. (2010). Murine hepatitis virus nonstructural protein 4 regulates virus-induced membrane modifications and replication complex function. J. Virol..

[17] Knoops K., Kikkert M., van den Worm S.H.E., Zevenhoven-Dobbe J.C., van der Meer Y., Koster A.J., Mommaas A.M., Snijder E.J. (2008). SARS-coronavirus replication is supported by a reticulovesicular network of modified endoplasmic reticulum. PLoS Biol..

[18] Hagemeijer M.C., Monastyrska I., Griffith J., van der Sluijs P., Voortman J., van Bergen en Henegouwen P.M., Vonk A.M., Rottier P.J.M., Reggiori F., de Haan C.A.M. (2014). Membrane rearrangements mediated by coronavirus nonstructural proteins 3 and 4. Virology.

[19] Mirdita M., Schütze K., Moriwaki Y., Heo L., Ovchinnikov S., Steinegger M. (2022). ColabFold: Making protein folding accessible to all. Nat. Methods.

[20] Jumper J., Evans R., Pritzel A., Green T., Figurnov M., Ronneberger O., Tunyasuvunakool K., Bates R., Žídek A., Potapenko A. (2021). Highly accurate protein structure prediction with AlphaFold. Nature.

[21] Varadi M., Anyango S., Deshpande M., Nair S., Natassia C., Yordanova G., Yuan D., Stroe O., Wood G., Laydon A. (2022). AlphaFold Protein Structure Database: Massively expanding the structural coverage of protein-sequence space with high-accuracy models. Nucleic Acids Res..

[22] Tunyasuvunakool K., Adler J., Wu Z., Green T., Zielinski M., Žídek A., Bridgland A., Cowie A., Meyer C., Laydon A. (2021). Highly accurate protein structure prediction for the human proteome. Nature.

[23] Uversky V.N. (2019). Intrinsically Disordered Proteins and Their “Mysterious” (Meta)Physics. Front. Phys..

[24] Mészáros B., Erdős G., Dosztányi Z. (2018). IUPred2A: Context-dependent prediction of protein disorder as a function of redox state and protein binding. Nucleic Acids Res..

[25] Jehl P., Manguy J., Shields D.C., Higgins D.G., Davey N.E. (2016). ProViz—A web-based visualization tool to investigate the functional and evolutionary features of protein sequences. Nucleic Acids Res..

[26] Hensel Z. (2022). Predicted binding interface between coronavirus nsp3 and nsp4. bioRxiv.

[27] Vriend G., Krause R., Hekkelman M.L., Nielsen J.E. The WHAT IF Web Interface. https://swift.cmbi.umcn.nl/servers/html/index.html.

[28] Krieger E., Vriend G. (2014). YASARA View-molecular graphics for all devices-from smartphones to workstations. Bioinforma. Oxf. Engl..

[29] Krieger E., Koraimann G., Vriend G. (2002). Increasing the precision of comparative models with YASARA NOVA—A self-parameterizing force field. Proteins Struct. Funct. Bioinforma..

[30] Bekker H., Berendsen H., Dijkstra E.J., Achterop S., Drunen R., van der Spoel D., Sijbers A., Keegstra H., Reitsma B., Renardus M.K.R. (1993). Gromacs: A parallel computer for molecular dynamics simulations. Phys. Comput..

[31] Abraham M.J., Murtola T., Schulz R., Páll S., Smith J.C., Hess B., Lindahl E. (2015). GROMACS: High performance molecular simulations through multi-level parallelism from laptops to supercomputers. SoftwareX.

[32] Pronk S., Páll S., Schulz R., Larsson P., Bjelkmar P., Apostolov R., Shirts M.R., Smith J.C., Kasson P.M., van der Spoel D. (2013). GROMACS 4.5: A high-throughput and highly parallel open source molecular simulation toolkit. Bioinforma. Oxf. Engl..

[33] Jorgensen W.L., Maxwell D.S., Tirado-Rives J. (1996). Development and Testing of the OPLS All-Atom Force Field on Conformational Energetics and Properties of Organic Liquids. J. Am. Chem. Soc..

[34] Robertson M.J., Tirado-Rives J., Jorgensen W.L. (2015). Improved Peptide and Protein Torsional Energetics with the OPLS-AA Force Field. J. Chem. Theory Comput..

[35] Lemkul J.A. (2019). From Proteins to Perturbed Hamiltonians: A Suite of Tutorials for the GROMACS-2018 Molecular Simulation Package [Article v1.0]. Living J. Comput. Mol. Sci..

[36] Lemkul J.A. Lysozyme in Water. http://www.mdtutorials.com/gmx/lysozyme/index.html.

[37] Verlet L. (1967). Computer “Experiments” on Classical Fluids. I. Thermodynamical Properties of Lennard-Jones Molecules. Phys. Rev..

[38] Essmann U., Perera L., Berkowitz M.L., Darden T., Lee H., Pedersen L.G. (1995). A smooth particle mesh Ewald method. J. Chem. Phys..

[39] Hess B., Bekker H., Berendsen H.J.C., Fraaije J.G.E.M. (1997). LINCS: A linear constraint solver for molecular simulations. J. Comput. Chem..

[40] Hess B. (2008). P-LINCS:  A Parallel Linear Constraint Solver for Molecular Simulation. J. Chem. Theory Comput..

[41] Bussi G., Donadio D., Parrinello M. (2007). Canonical sampling through velocity rescaling. J. Chem. Phys..

[42] Parrinello M., Rahman A. (1981). Polymorphic transitions in single crystals: A new molecular dynamics method. J. Appl. Phys..

