# Supplementary material for: Modelling the Transitioning of SARS-CoV-2 nsp3 and nsp4 Lumenal Regions towards a More Stable State on Complex Formation
Source: Int J Mol Sci. 2022 Dec 31;24(1):720. doi: 10.3390/ijms24010720 (PMC9821074; doi:10.3390/ijms24010720)
Supplement: Supplementary file 1 [file ijms-24-00720-s001.zip › ijms-2033318-supplementary.pdf]

Supplementary Materials

Modelling the Transitioning of SARS-CoV-2 nsp3 and nsp4 Lumenal Regions towards a More Stable State on Complex Formation

Nele Klatte [1], Denis C. Shields [1,2,*] and Clement Agoni [1,2,3]

[1] UCD Conway Institute of Biomolecular and Biomedical Research, University College Dublin, Belfield D04 V1W8, Ireland
[2] School of Medicine, University College Dublin, Belfield D04 V1W8, Ireland
[3] Discipline of Pharmaceutical Sciences, School of Health Sciences, University of KwaZulu Natal, Durban 4041, South Africa
* Correspondence: denis.shields@ucd.ie

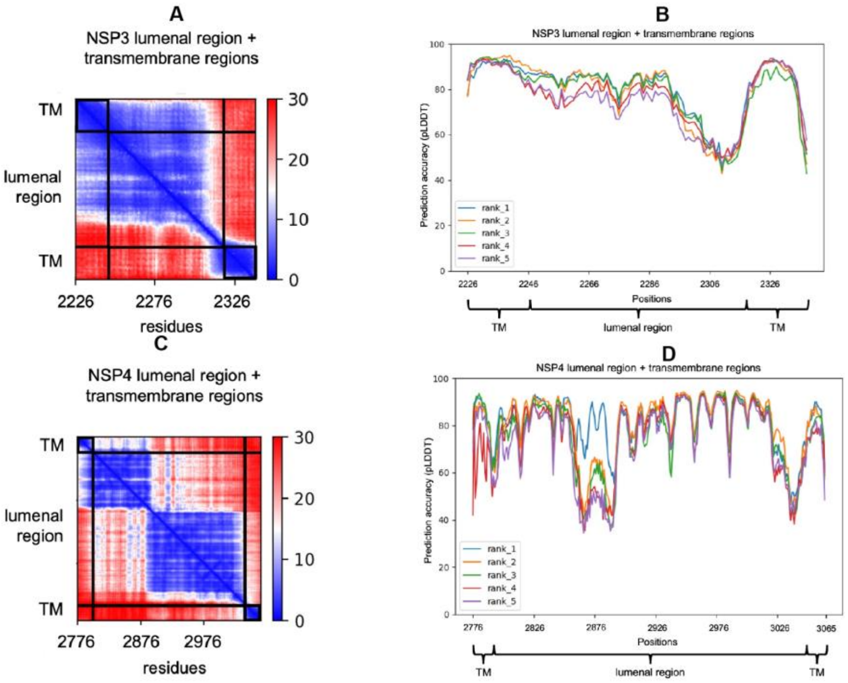

Figure S1. Quality assessment plots of the nsp3 lumenal region with adjoining transmembrane regions and the nsp4 lumenal region with adjoining transmembrane regions. (A) PAE plot of the highest-ranked model of the nsp3 lumenal region with adjoining transmembrane regions. (B) pLDDT plot of the five models of the nsp3 lumenal region with adjoining transmembrane regions. (C) PAE plot of the highest-ranked model of the nsp4 lumenal region with adjoining transmembrane regions. (D) pLDDT plot of the five models of the nsp4 lumenal region with adjoining transmembrane regions.

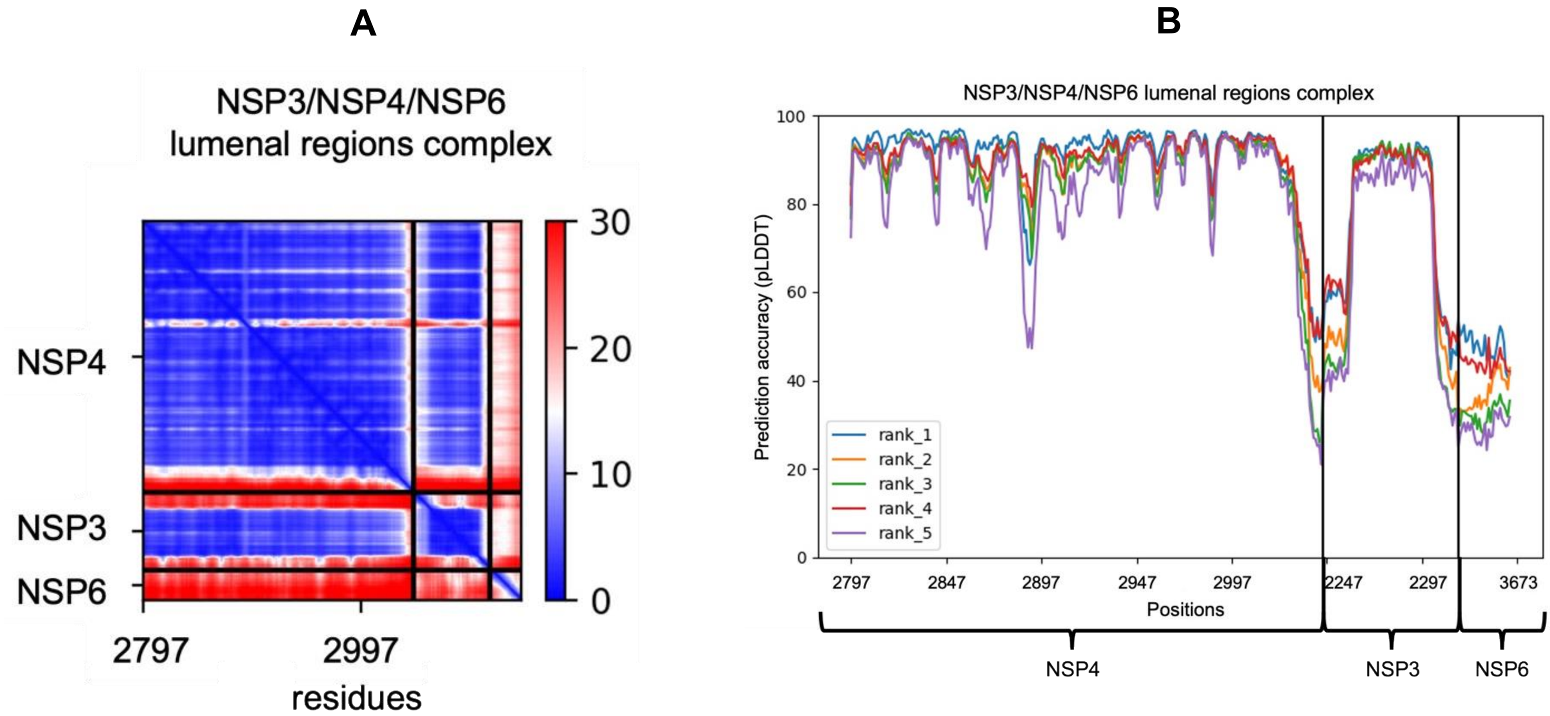

Figure S2. Quality assessment plots of the nsp3, nsp4 and nsp6 lumenal regions in complex (A) PAE plot of the highest-ranked model of the nsp3, nsp4 and nsp6 lumenal regions in complex. (B) pLDDT plot of the five models of the nsp3, nsp4 and nsp6 lumenal regions in complex.

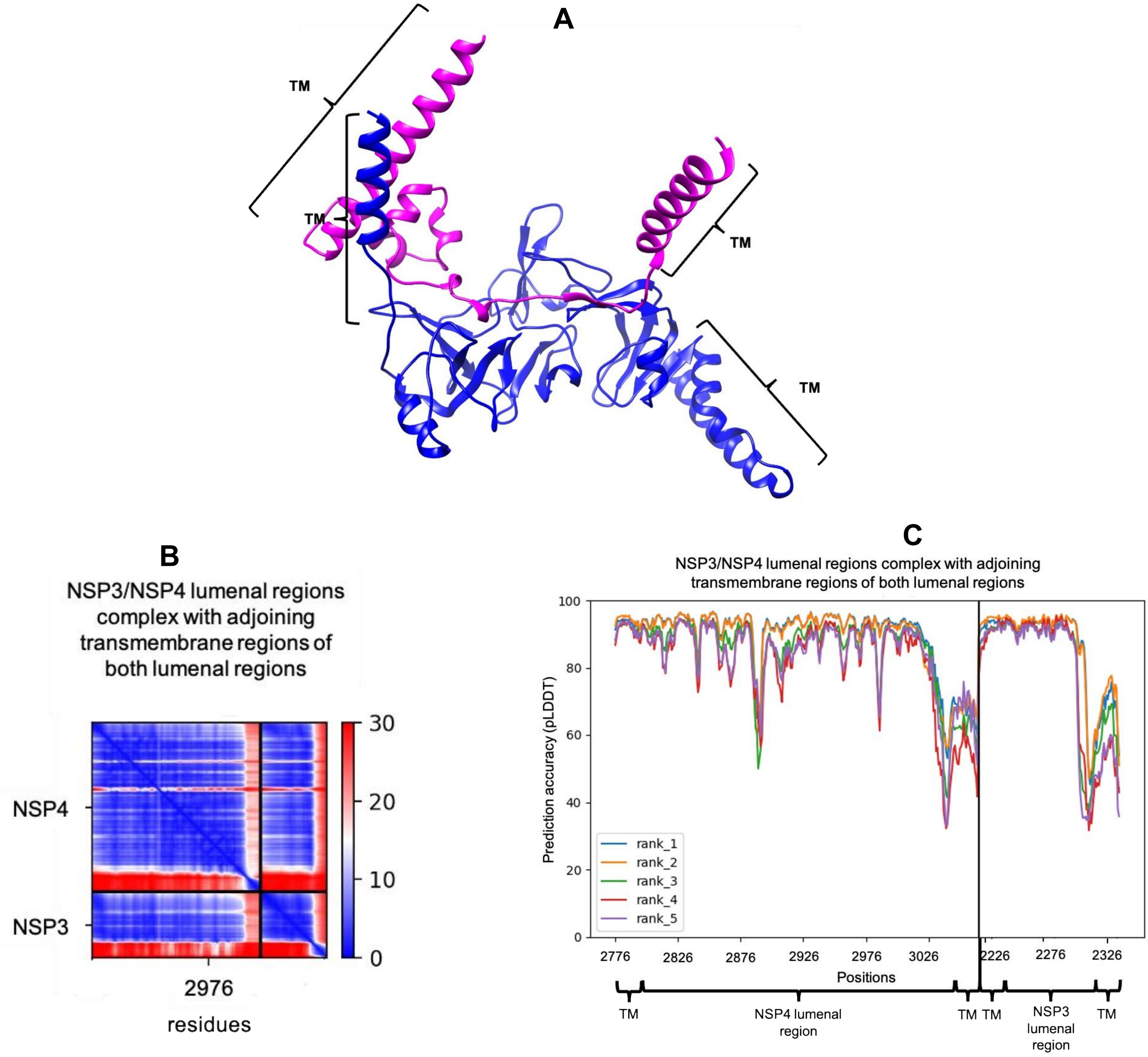

Figure S3. Structure of highest ranked models and quality assessment plots of the nsp3 and nsp4 lumenal regions complex with adjoining transmembrane regions of both lumenal regions. (A) Structure of the highest-ranked model of the nsp3 (magenta) and nsp4 (blue) lumenal regions complex with adjoining transmembrane regions of both lumenal regions. (B) PAE plot of the highest-ranked model of the nsp3 and nsp4 lumenal regions complex with adjoining transmembrane regions of both lumenal regions. (C) pLDDT plot of the five models of the nsp3 and nsp4

lumenal regions complex with adjoining transmembrane regions of both lumenal regions.

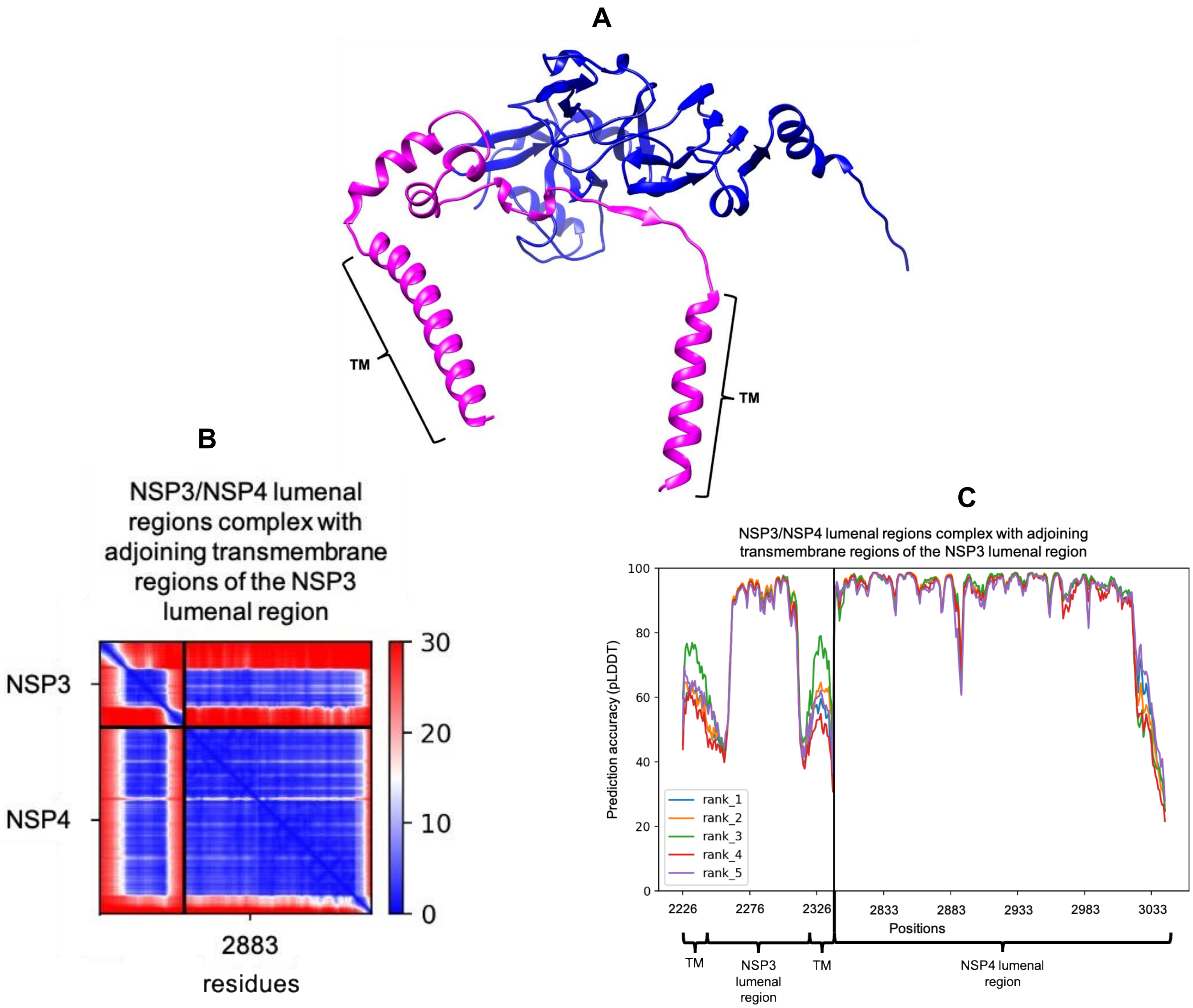

Figure S4. Structure of highest ranked models and quality assessment plots of the nsp3 and nsp4 lumenal regions complex with adjoining transmembrane regions of the nsp3 lumenal region. (A) Structure of the highest-ranked model of the nsp3 (magenta) and nsp4 lumenal (blue) regions complex with adjoining transmembrane regions of the nsp3 lumenal region. (B) PAE plot of the highest-ranked model of the nsp3 and nsp4 lumenal regions complex with adjoining transmembrane regions of the nsp3 lumenal region. (C) pLDDT plot of the five models of the nsp3 and nsp4 lumenal regions complex with adjoining transmembrane regions of the nsp3 lumenal region.

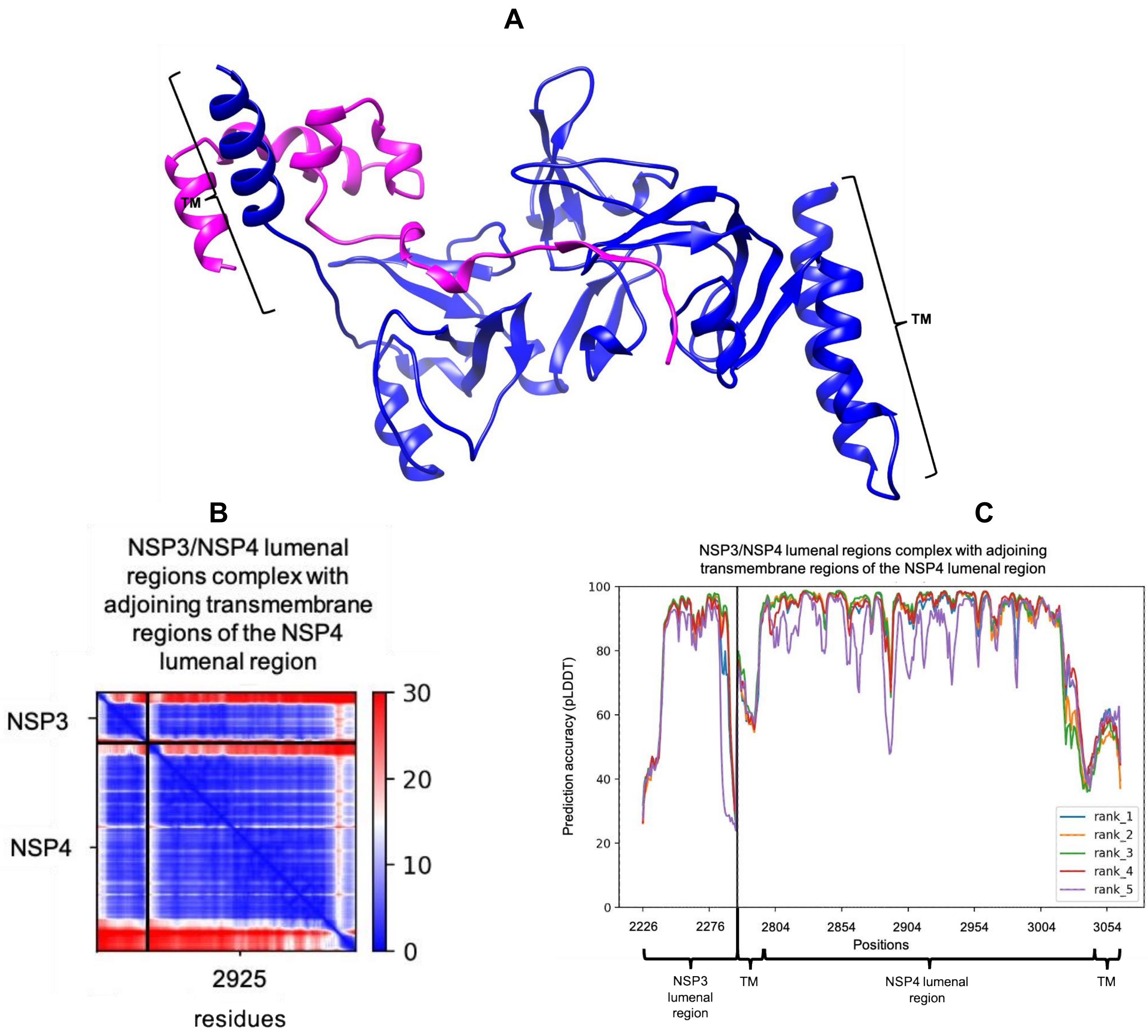

Figure S5. Structure of highest ranked models and quality assessment plots of the nsp3 and nsp4 lumenal regions complex with adjoining transmembrane regions of the nsp4 lumenal region. (A) Structure of the highest-ranked model of the nsp3 (magenta) and nsp4 (blue) lumenal regions complex with adjoining transmembrane regions of the nsp4 lumenal region. (B) PAE plot of the highest-ranked model of the nsp3 and nsp4 lumenal regions complex with adjoining transmembrane regions of the nsp4 lumenal region. (C) pLDDT plot of the five models of the nsp3

and nsp4 lumenal regions complex with adjoining transmembrane regions of the nsp4 lumenal region.

Table S1. Potential energies of the nsp3 lumenal region during 100 ns of MD simulation.

Simulation time [ns]	Potential energy [kJ/mol]
10	-1655.59
20	-1690.33
30	-1592.00
40	-1634.21
50	-1663.38
60	-1706.94
65	-1705.19
68	-1705.34
70	-1713.08
72	-1701.85
75	-1711.93
77	-1726.08
78	-1718.68
80	-1705.70
90	-1700.80
100	-1682.93

Table S2. Potential energies of the nsp4 lumenal region during 150 ns of MD simulation.

Simulation time [ns]	Potential energy [kJ/mol]
10	-8301.60
12	-8266.04
15	-8275.47
20	-8237.44
30	-8186.22
40	-8226.25

50	-8251.56
60	-8208.09
63	-8209.83
65	-8265.35
67	-8196.40
70	-8257.28
75	-8209.16
80	-8204.33
85	-8262.10
90	-8253.14
100	-8227.99
110	-8279.06
120	-8256.03
125	-8275.15
128	-8307.16
130	-8339.95
132	-8309.59
135	-8169.12
140	-8195.24
145	-8299.13
148	-8330.72
150	-8345.94

Table S3. Potential energies of the nsp3 and nsp4 lumenal regions in complex during 100 ns of MD simulation.

Simulation time [ns]	Potential energy [kJ/mol]
10	-10150.42
20	-10087.05
30	-10148.12
40	-10256.76
50	-10227.05

60	-10252.95
70	-10262.68
80	-10224.53
83	-10255.17
85	-10279.55
87	-10209.55
90	-10271.49
95	-10213.36
100	-10220.22

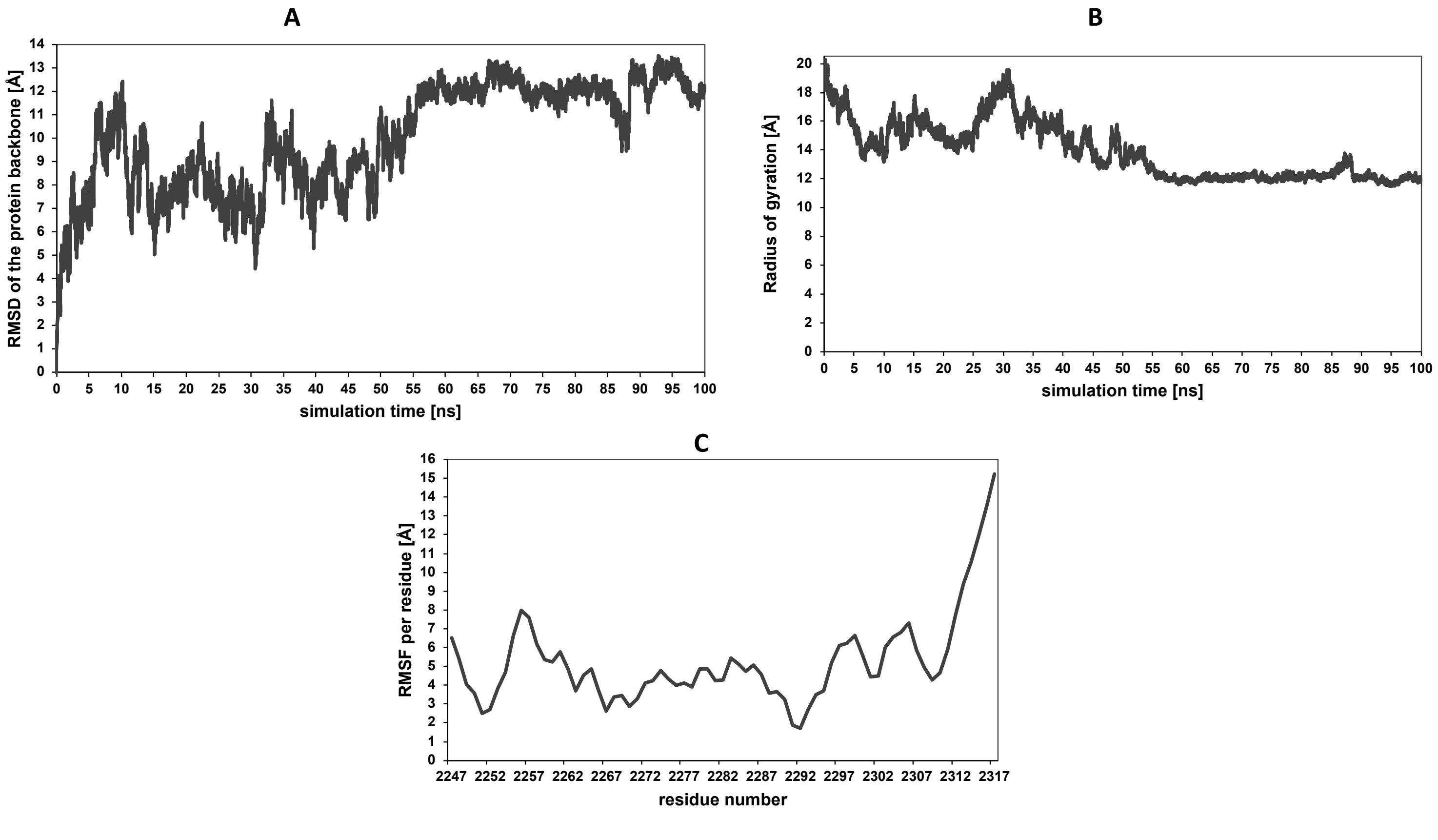

Figure S6. Analysis plots of the nsp3 lumenal region during 100 ns of MD simulation. (A) RMSD of the protein backbone. (B) Radius of gyration. (C) RMSF per residue.

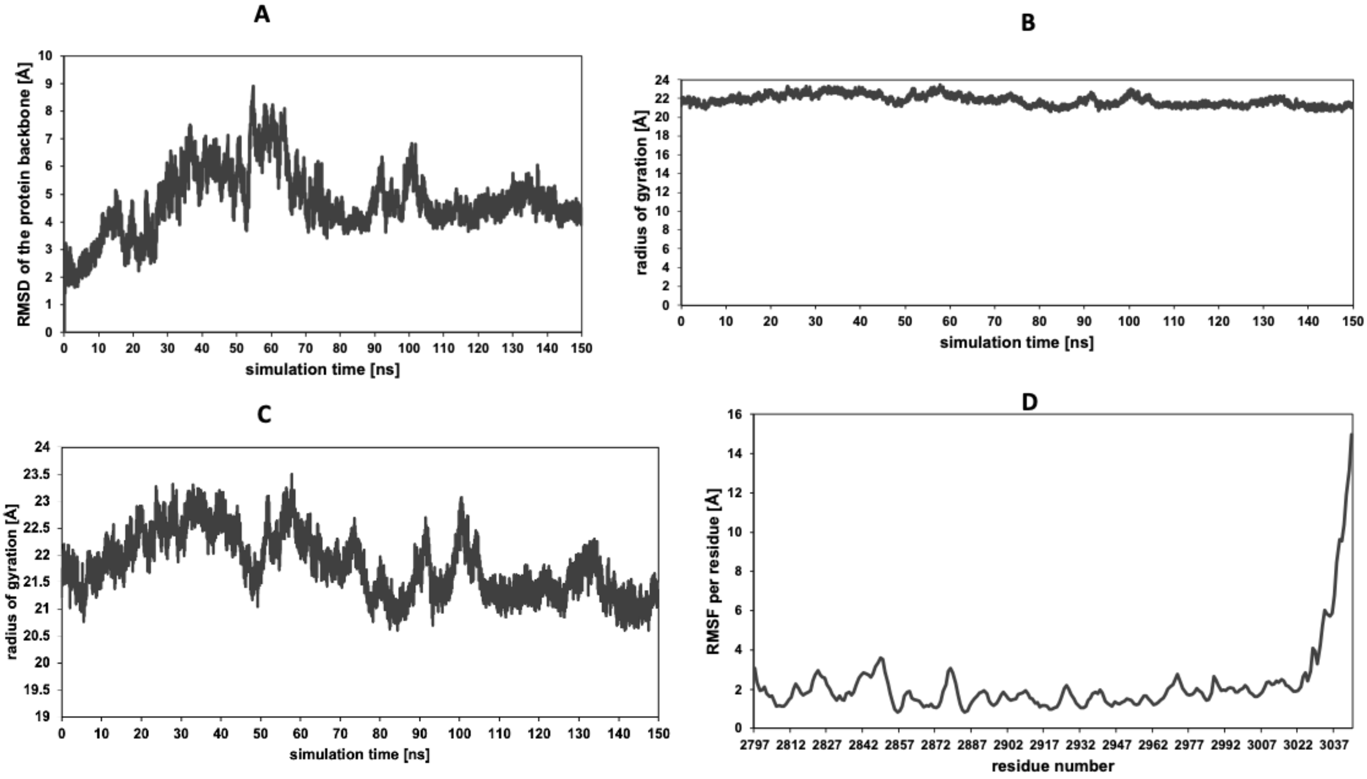

Figure S7. Analysis plots of the nsp4 lumenal region during 150 ns of MD simulation. (a) RMSD of the protein backbone. (b) Radius of gyration. (c) Radius of gyration (zoomed in). (d) RMSF per residue.

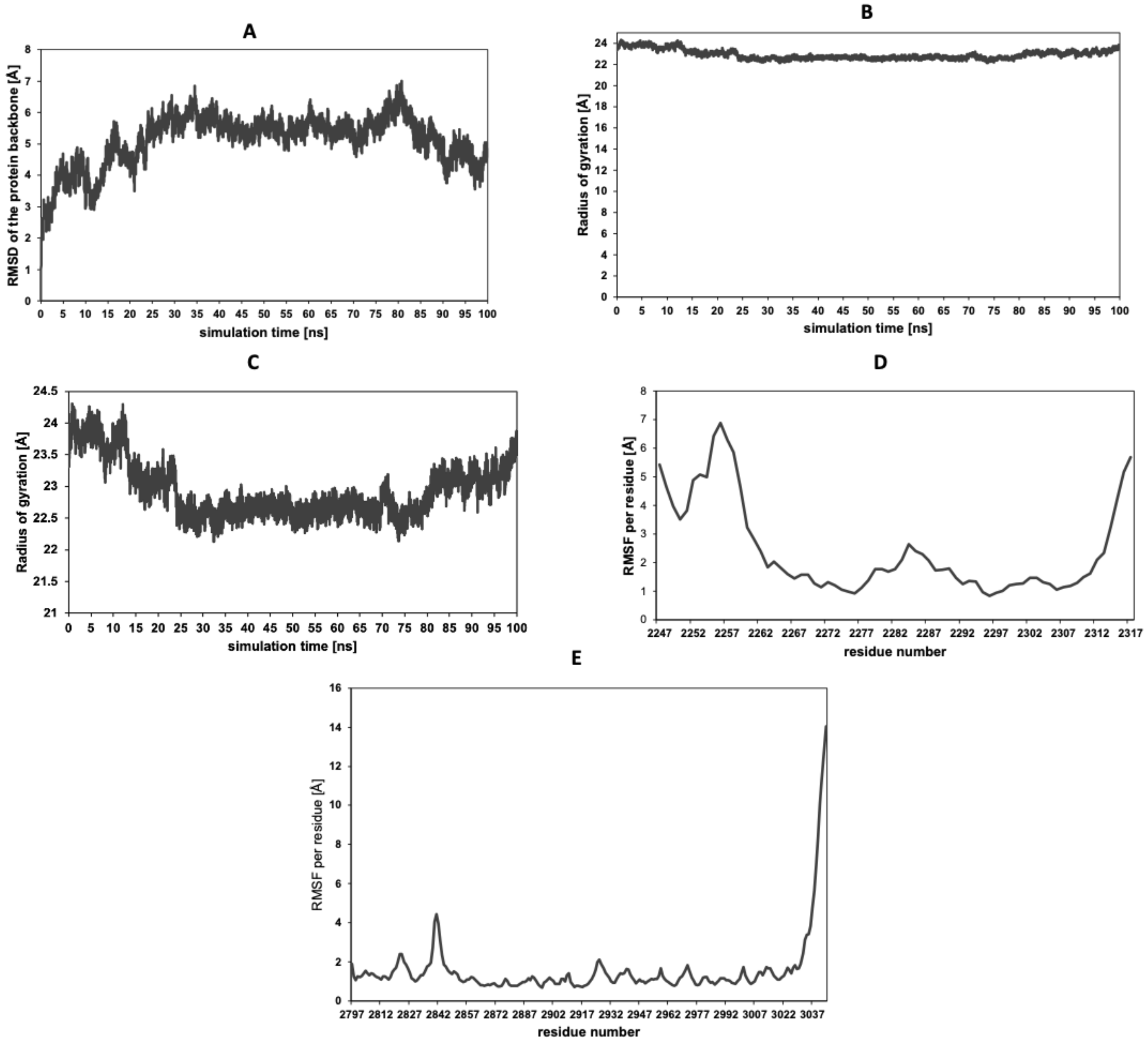

Figure S8. Analysis plots of the nsp3 and nsp4 lumenal regions in complex during 100 ns of MD simulation. (a) RMSD of the protein backbones. (b) Radius of gyration. (c) Radius of gyration (zoomed in). (d) RMSF per residue of the nsp3 lumenal region. (e) RMSF per residue of the nsp4 lumenal region.

Table S4. Input sequences of the protein fragments used for protein structure prediction with ColabFold.

Description	Uniprot identifier	Sequence range	Sequence
nsp3 lumenal region	P0DTD1	2247-2317	TAALGVLMSNLGMPSYCTGYREGYLNSTNVTIATYCTGSIPCSVCLSGLDSLDTYPSLETIQITISSFKWD
nsp4 lumenal region	P0DTD1	2797-3044	SKHTDFSSEIIGYKAIDGGVTRDIASTDTCFANKHADFDTWFSQRGGSYTNDKACPLIAAVITREVGFVVPGLPGTILRTTNGDFLHFLPRVFSAVGNICYTPSKLIEYTDFATSACVLAAECTIFKDASGKPVPY

			CYDTNVLEGSVAYESLRPDTRYVLMDGSIIQFPNTYLEGSVRVVTTFDSEYCRHGTCERSEAGVCVSTSGRWVLNNDYYRSLPGVFCGVDAVNLLTNMFTPLIQPIGALDIS
nsp3 lumenal region with adjoining transmembrane regions	P0DTD1	2226-2338	LINIIIWFLLLSVCLGSLIYSTAALGVLMSNLGMPSYCTGYREGYLNSTNVTIATYCTGSIPCSVCLSGLDSLDTYPSLETIQITISSFKWDLTAFGLVAEWFLAYILFTRFF
nsp4 lumenal region with adjoining transmembrane regions	P0DTD1	2776-3065	VTLVFLFVAAIFYLITPVHVMSKHTDFSSEIIGYKAIDGGVTRDIASTDTCFANKHADFDTWFSQRGGSYTNDKACPLIAAVITREVGFVVPGLPGTILRTTNGDFLHFLPRVFSAVGNICYTPSKLIEYTDFATSACVLAAECTIFKDASGKPVPYCYDTNVLEGSVAYESLRPDTRYVLMDGSIIQFPNTYLEGSVRVVTTFDSEYCRHGTCERSEAGVCVSTSGRWVLNNDYYRSLPGVFCGVDAVNLLTNMFTPLIQPIGALDISASIVAGGIVAIVVTCLAYYFM
nsp6 lumenal region	P0DTD1	3656-3673	PASWVMRIMTWLDMVDTS

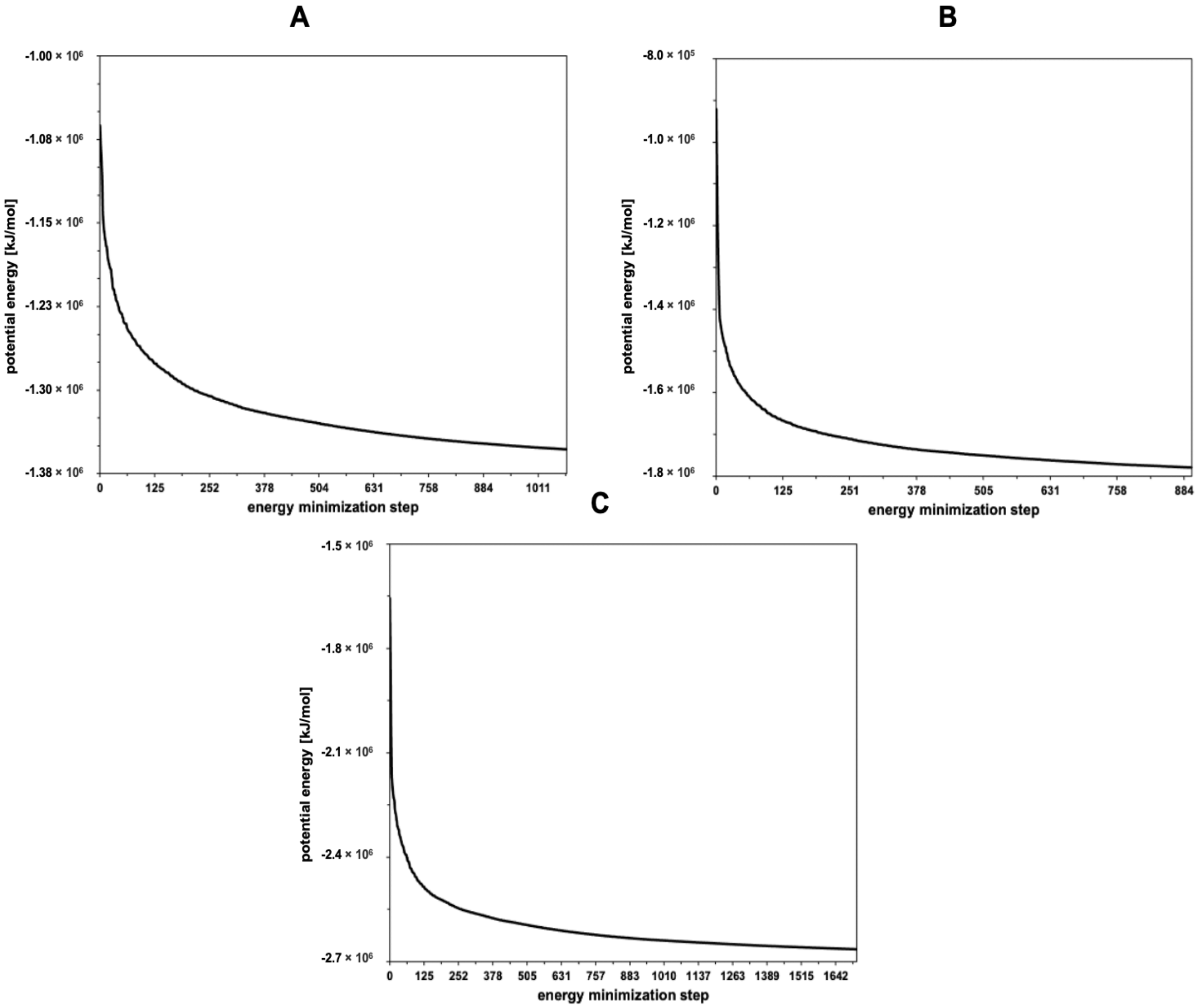

Figure S9. Potential energies of the simulated structures during energy minimisation before MD simulation. (a) nsp3 lumenal region. (b) nsp4 lumenal region. (c) nsp3 and nsp4 lumenal regions in complex.
